# Peritoneal Leiomyoma in a Male Patient: A Case Report

**DOI:** 10.7759/cureus.68100

**Published:** 2024-08-29

**Authors:** Vraj Patel, Harshal Patel, Mark Wall, John J Nelson, Scott Brashier

**Affiliations:** 1 Radiology, William Carey University College of Osteopathic Medicine, Hattisburg, USA; 2 General Surgery, William Carey University College of Osteopathic Medicine, Hattisburg, USA; 3 Radiology, Merit Health Biloxi, Biloxi, USA; 4 Pathology, Merit Health Biloxi, Biloxi, USA; 5 General Surgery, Merit Health Biloxi, Biloxi, USA

**Keywords:** lower abdominal pain, smooth muscle tumor, a case report, mesenteric hematoma, peritoneal leiomyoma

## Abstract

Leiomyomas are smooth muscle tumors that are commonly present in premenopausal women. These tumors are benign and of monoclonal origin. Peritoneal cavity leiomyomas are commonly reported in females and rarely reported in males. Here, we present a 58-year-old male who presented to the emergency room with abdominal pain. Computed tomography (CT) scan of the abdomen revealed multiple well-circumscribed left lower quadrant mesenteric masses containing heterogeneous attenuation and macroscopic fat. Exploratory laparotomy performed following abdominal CT and subsequent CT-guided biopsy revealed two intra-abdominal masses. Histopathological evaluation was positive for desmin and caldesmon immunohistochemical stains, and negative for C-kit, consistent with benign leiomyomata. This case highlights a benign leiomyoma within the abdominal cavity, which is an extremely rare occurrence and a potentially rare cause of abdominal pain.

## Introduction

Leiomyomas are benign neoplasms or smooth muscle origin. Leiomyomas are common benign tumors of the reproductive tract and are present in 20% of women of reproductive age [1]. However, rare gastrointestinal tumors of mesenchymal origin can occur such as leiomyomas, leiomyosarcomas, peripheral nerve sheath tumors, and much more [2]. Benign soft tissue neoplasms are more common; however, malignant mesenchymal neoplasms like gastrointestinal stromal tumors (GISTs) are important to evaluate. GISTs arise from interstitial cells of Cajal as a result of a C-kit proto-oncogene mutation [3]. The most common locations for GIST are the stomach (50%-60%) and small bowel (20%-30%). Prognostic factors include mitotic activity, tumor size, and anatomic site. There is also a significant risk of dissemination due to intraoperative tumor rupture [4]. Hence, it is important to evaluate and confirm these abdominal leiomyomas of gastrointestinal origin to rule out metastatic disease. Abdominal cavity leiomyomas are exceedingly rare and are typically thought to follow surgical resection of uterine fibroids [5]. Treatment consists of the complete removal of these benign smooth muscle growths. Abdominal leiomyomas are more common in women. In this article, we report a case of multiple abdominal leiomyomas in a middle-aged male with no significant medical history.

## Case presentation

A 58-year-old male with no significant past medical history presented to the emergency room with new-onset abdominal pain. He described the abdominal pain as sharp and severe in the mid-abdomen and left lower quadrant. His family history was noncontributory. Physical exam at the time of initial presentation was negative for peritoneal signs or other acute abdominal findings. Following conservative outpatient management, his abdominal pain persisted, and he was further evaluated with referral to interventional radiology for computed tomography (CT)-guided percutaneous biopsy for definitive diagnosis before further management decisions.

**Figure 1:**
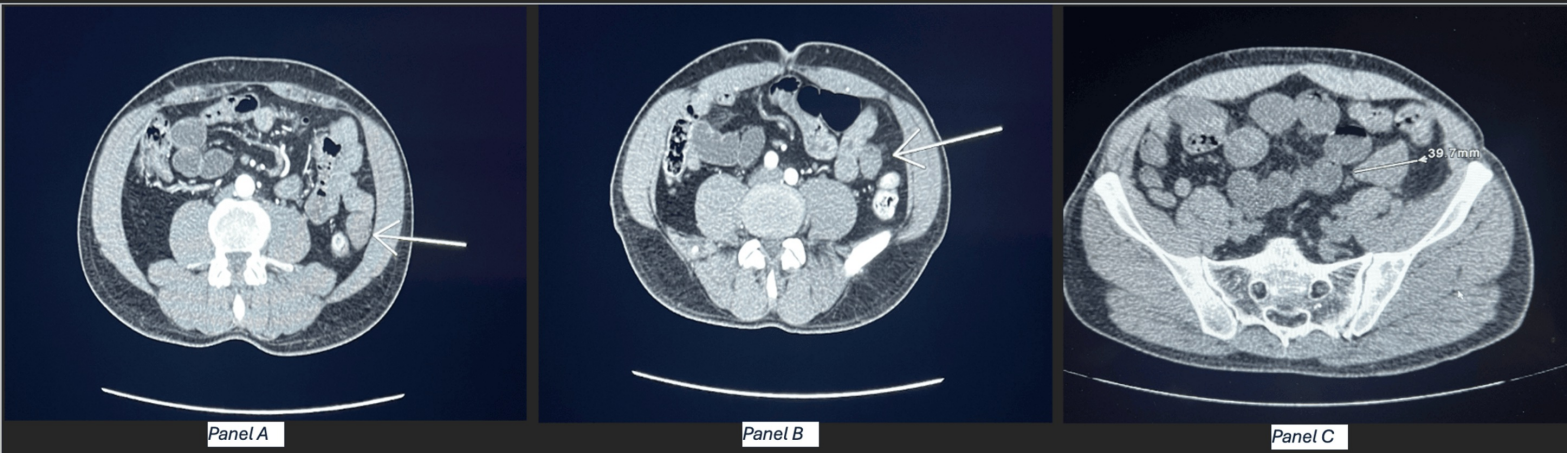
CT scan with panels A, B, and C (left to right) Panel A: Axial CT of the abdomen. The arrow points to mass A in the left lower quadrant. Panel B: Axial CT of the abdomen. The arrow points to mass B in the left lower quadrant. Panel C: Axial CT of the abdomen. Measurement of mass C in the left lower quadrant is shown.

On presentation before the percutaneous biopsy, the patient was awake, alert, oriented, and in a normal state of health. CT scan of the abdomen revealed multiple left lower quadrant mesenteric masses containing heterogeneous attenuation including macroscopic fat (Figure 1).

**Figure 2:**
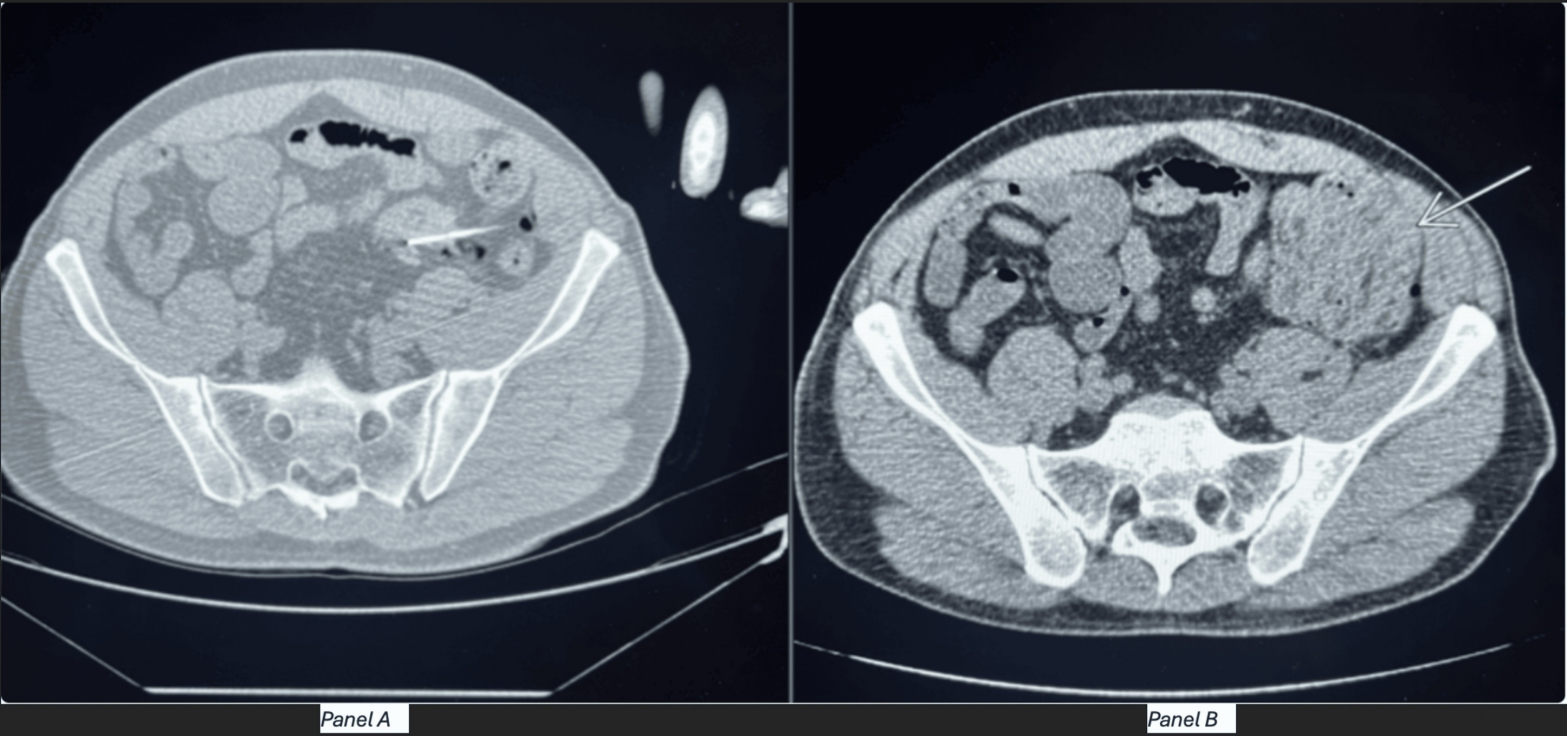
CT scan taken during (panel A) and after (panel B) the biopsy. Panel A: CT-guided needle biopsy. Panel B: Arrow pointing to subsequent hematoma formation following CT-guided needle biopsy.

A CT-guided needle biopsy was performed. Following successful 18-gauge core biopsy acquisition, the patient reported left lower quadrant pain and the post-biopsy CT imaging revealed a developing left lower quadrant hematoma and a small amount of hemoperitoneum tracking in the left pericolic gutter (Figure 2). Initial vitals were stable following the biopsy; however, after 15 minutes of observation, the patient's blood pressure decreased and heart rate elevated. The decision was made to transfer the patient to the emergency room for observation and urgent care. The patient reported worsening abdominal pain upon which a general surgery consultation was placed. The abdominal exam was significant for firm diffuse tenderness on palpation and rebound tenderness. Physical examination and declining state thus warranted a diagnostic laparoscopy for control of internal hemorrhage.

**Figure 3:**
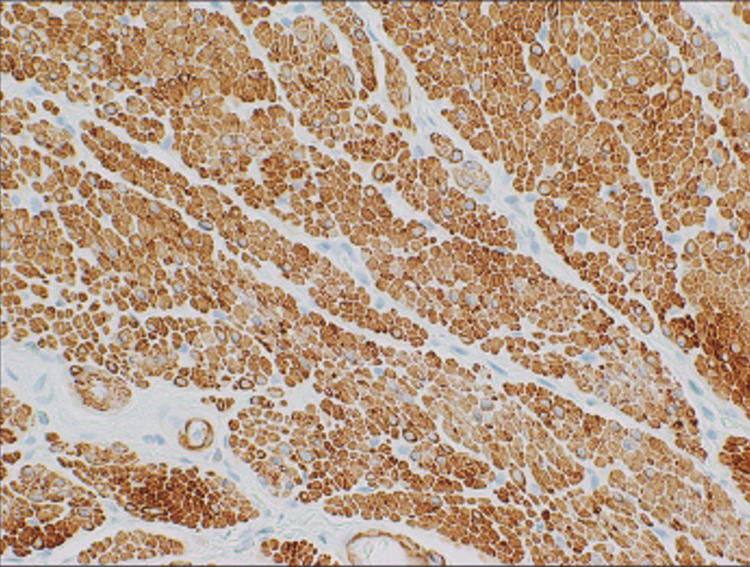
Leiomyoma 40x, caldesmon The caldesmon-stained image highlights the presence and distribution of smooth muscle cells, indicated by the brown staining.

**Figure 4:**
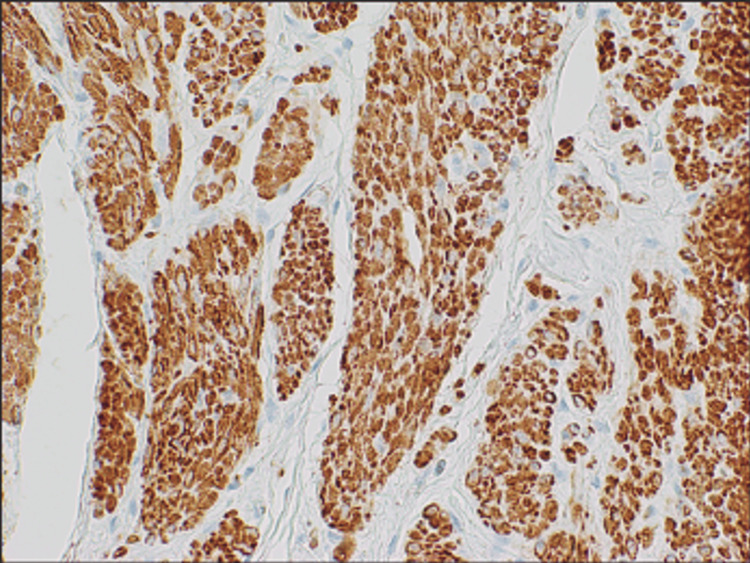
Leiomyoma 40x, desmin The desmin-stained image highlights muscle cells, with brown stains indicating the presence of desmin protein.

**Figure 5:**
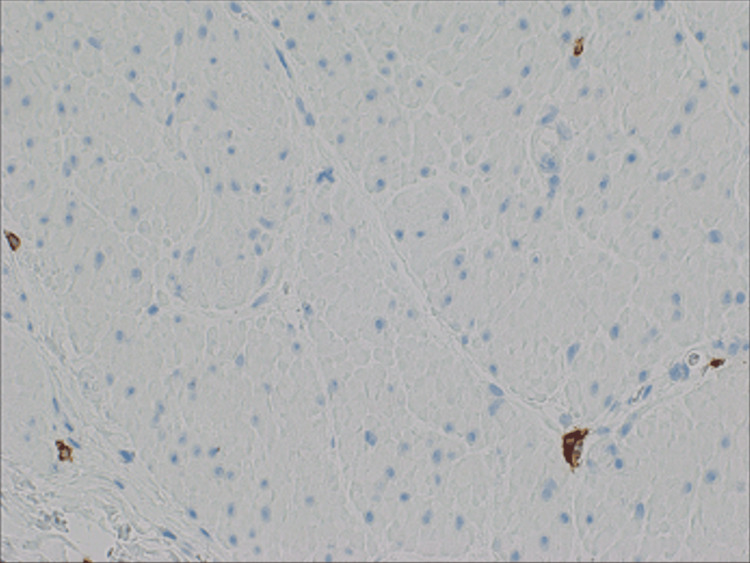
Leiomyoma 40x, C-kit negative The figure shows the absence of cytoplasmic staining/C-kit (CD117) negative.

The initial diagnosis upon pathological evaluation was a mesenchymal tumor with neural features favoring ganglioneuroma. The lesion was deemed relatively rare, and an expert consultation was requested. Upon further evaluation, immunohistochemistry was positive for desmin and caldesmon (Figures 3, 4) and negative for C-kit (Figure 5), which led to the diagnosis of benign leiomyomata.

Following the expansion of hematoma, the patient was brought to the operating room on an urgent basis. Diagnostic laparoscopy revealed hemoperitoneum in the paracolic gutters tracking down to the pelvis. A total of 500 cc of blood was suctioned during the investigation of the mesenteric hematoma. The hematoma was incompressible with poor visualization of active bleeding below the hematoma. An intraoperative decision was made to convert to an open exploratory laparotomy to allow for better visualization and treatment for evacuating the hematoma. Midline incision was made followed by elevation of the fascia and peritoneum. A mesenteric window was created, which allowed for the hematoma evacuation, resulting in the evacuation of 500 cc more blood. Following the evacuation of the hematoma, a small bleeding mesenteric artery was identified and suture-ligated. Two intra-abdominal 4 cm masses were found medial to the colon and anterior to the mesentery consistent with findings described on the CT scan. Masses were excised and sent to pathology. A third mass was also found superiorly, but surgical excision was not feasible. A clinical decision was made to leave the mass and monitor it periodically with CT for growth. The abdomen was irrigated and closed. A Jackson-Pratt (JP) drain was placed exiting the right lower quadrant and anchored to the skin. The immediate postoperative course was routine without complications.

The patient did well during his postoperative course. The total length of hospitalization following surgery was five days. Initial postoperative pain improved significantly during his stay. His JP drain contained some dark bloody outputs throughout his stay; however, his hemoglobin and hematocrit remained stable. Throughout this postoperative stay, his diet was advanced to a normal diet. His JP drain output became serous, and the drain was removed before discharge.

Following normal postoperative recovery and improved abdominal pain symptoms, a plan was established for yearly CT follow-up. The decision was made to follow the remaining abdominal mass in lieu of additional surgical resection due to his improved symptoms. Although the patient has improved, he reports periodic left lower quadrant pain. A year after the patient’s surgery, his leiomyoma was 4.7 x 2.3 cm, which had slightly enlarged since the previous year when it measured 4.3 x 2.1 cm. The patient was adherent to his CT screenings, and there have been no adverse or unanticipated events. He reports periodic but tolerable abdominal discomfort and continues to elect for annual surveillance.

## Discussion

​​Leiomyomas are common benign smooth muscle tumors present in women; however, they are very rare to be found in the abdominal cavity in a middle-aged male. Gastrointestinal tumors have been reported, and esophageal leiomyomas are the most common esophageal benign tumors [1]. Neoplasms such as GIST are also part of the differential and must be ruled out. GISTs originate from interstitial cells of Cajal and arise due to a C-kit mutation [5]. CD117, a C-kit proto-oncogene product is expressed by interstitial cells of Cajal, and a majority of GISTs express this product; hence, it is important to evaluate histologically [6]. Prior reports of abdominal leiomyomas show characteristic staining of smooth muscle tumors such as desmin and smooth muscle actin (SMA) in addition to CD117, estrogen receptor (ER), and progesterone receptor (PR) [7].

Abdominal leiomyomas in males occur in an age range of 22-45 years at the time of diagnosis [4]. Previously reported cases discussed much larger tumor sizes than the one found in our patient. In the case report of Wang et al., the 22-year-old male presented with solely abdominal distension due to an abdominal leiomyoma at the size of 30 x 19 x 33 cm [7]. Another case report presented a 24-year-old male with the complaint of a large epigastric mass and a two-month history of poor appetite with 15 kg weight loss; this patient’s leiomyoma was 25 x 21 x 11 cm [8]. Presenting signs and symptoms are due to the large size of the tumor obstructing nearby intra-abdominal structures or causing abdominal distension. As our patient's tumors were 4.7 x 2.3 cm and 4.3 x 2.1 cm, they may not have caused any obstruction, pain, or distension until the tumor grew and caused pain. This may explain the older age of 58 at the time of diagnosis.

It is certainly a limitation and challenge to draw conclusions and causations of such pathology as there is very limited reported clinical data in men. However, a potential strength is that highlighting such rare cases provides the opportunity for earlier detection, management, and awareness of abdominal leiomyomas in males.

## Conclusions

Abdominal cavity leiomyomas are a rare cause of abdominal pain presenting in male patients. This case demonstrates that although extremely rare, leiomyomas can be found within the mesentery and can be symptomatic depending on the location and size of these smooth muscle tumors. The current mainstay treatment is surgical resection for symptomatic abdominal leiomyomas with surveillance as a reasonable approach for asymptomatic patients and patients with mild abdominal discomfort.
